# Some thoughts and greetings from the new Editor-in-Chief

**DOI:** 10.1007/s11547-020-01324-6

**Published:** 2021-01-27

**Authors:** Antonio Barile

**Affiliations:** Department of Biotechnology and Applied Clinical Sciences, University of L’Aquila, L’Aquila, 67100 Italy

It will probably seem obvious to readers but, first of all, I want to tell you that I am truly honored and happy to have been selected as the new Editor-in-Chief of “La Radiologia Medica” (RAME).

For this, I want to thank the Board of Directors of the Italian Society of Medical and Interventional Radiology (SIRM) and, above all, the EIC that preceded me, Prof. Andrea Giovagnoni, who strongly wanted me in his editorial staff in 2018 and who taught me to respect and love this job. As John of Salisbury writes in his “Metalogicon” of 1159: “…dicebat Bernardus Carnotensis nos esse quasi nanos, gigantium humeris insidentes, ut possimus plura eis et remotiora videre, non utique proprii visus acumine, aut eminentia corporis, sed quia in altum subvenimur et extollimur magnitude gigantea”.

Grazie di cuore Andrea!!!

In this new era of RAME, there are a number of changes that I would like to highlight to the journal’s readers, as we are confident that such changes will appeal to a wide range of both academic and clinical interests.

The excellent work done by those who preceded me has produced in the last year an important increase in journal performance across various metrics. The future goal will be to further increase the ranking of RAME, and this will necessarily require an editorial strategy that includes various initiatives designed to further increase the quality of papers contributions, attracting researchers from across the international academic community, to increase the number of citations and the overall performance of the journal.

As the Editor-in-Chief, I recognize the value authors place on high-quality, impartial peer review conducted in a timely manner. Furthermore, it is extremely important to have a quick publication and, to this end, we will try to better structure our editorial team in order to speed up the processing of the submitted manuscripts. I have educated all those already involved with the journal in an effort to provide the highest standards of manuscript review, modification and publication. We have implemented strict peer review criteria, and this will affect the quality of published articles.

In updating the Editorial Board, I will therefore try to select a balanced global representation of the leaders in the various sectors of diagnostic and interventional radiology in order to have an editorial team fully committed to the success of this extraordinary journal.

I would also like to introduce and develop, as we are living in a technological era, a social communication service that allows those who follow us to be always updated on the editorial news of the journal, and because of this, I really believe that Twitter may be the right way to go.

I also invite talented researchers within our international academic community to continue supporting RAME by submitting their research for publication and playing an active role in the review process.

It will therefore be a pride and an honor for me to be able to work with an exceptional team of associate editors, deputy editors and all members of the editorial board.

Finally, let me thank our publisher Springer, and their devoted team of experts, for their outstanding work, guidance and encouragement for this new adventure.

Thank you all once again for your extraordinary support and continued efforts to ensure that “La Radiologia Medica” is recognized as one of the leading journals in its field.

